# Editorial: Debates in Clinical Management in Pediatric Endocrinology

**DOI:** 10.3389/fendo.2021.663860

**Published:** 2021-03-10

**Authors:** Maria Loredana Marcovecchio, Barbara Predieri, Gianpaolo De Filippo, Maurizio Delvecchio

**Affiliations:** ^1^ Department of Paediatrics, University of Cambridge, Cambridge, United Kingdom; ^2^ Pediatric Unit, Department of Medical and Surgical Sciences of the Mother, Children and Adults, University of Modena and Reggio Emilia, Modena, Italy; ^3^ Assistance Publique - Hôpitaux de Paris, Service d'Endocrinologie et Diabétologie Pédiatrique, Hôpital Robert Debré, Paris, France; ^4^ Metabolic and Genetic Disorders, “Giovanni XXIII” Children’s Hospital, Bari, Italy

**Keywords:** endocrinology and diabetes, puberty, hypogonadism, small for gestational age (SGA), bone maturation, bone age, glucokinase (GCK)

## Introduction

Over the last decades the diagnosis and management of pediatric endocrine disorders has substantially improved. However, clinicians still face some key challenges, without straightforward solutions, during their daily clinical practice.

This Research Topic includes a collection of papers on relevant topics in Pediatric Endocrinology and Diabetes, which can support clinicians in better understanding endocrine conditions and their management.

The global obesity epidemic has brought to light that metabolic complications, previously thought to affect only obese adults, can manifest at a younger age. The true incidence of pediatric type 2 diabetes (T2D) is still debated (1), and North American and European studies show contrasting results. Indeed, the exponential increase in the incidence of T2D reported among North American obese children and adolescents is not confirmed among European youth. Data from different populations and Countries are essential to better understand the epidemiology of T2D. In this issue, Al-Kandari et al. report the incidence of T2D in Kuwait, providing some Country-specific unique epidemiological data.

The prevalence of glucokinase (GKC) deficit in pregnant women with diabetes is the focus of Bitterman et al.’s paper. Although maturity onset diabetes of the young (MODY) due to GCK deficit is a benign condition, a prompt diagnosis is essential for the optimal course of pregnancy and planning of appropriate newborn care. Available data indicate that the prevalence of GKC deficit varies between 0-80% among pregnant women, dependent on the screening criteria and the populations’ characteristics (2, 3). In this retrospective study, the authors show that even the recent “new-pregnancy-specific criteria,” based on BMI <25 kg/m^2^ and fasting glycemia >99 mg/dl, do not reach optimal sensitivity and specificity thresholds. Therefore, better criteria applicable to different populations, are required for a tailored selection of pregnant women eligible for genetic tests.

This issue also includes a systematic review and meta-analysis addressing the potential association between type 1 diabetes (T1D) and periodontal disease in children (4). Previous studies reported a three- to four-fold increased risk of periodontal disease in adults with T2D and suggested a bidirectional relationship between these two conditions. Rapone et al. highlight lack of strong evidence for similar associations in children with T1D. Current evidence is primarily based on cross-sectional studies, which show a higher prevalence of periodontal disease in children with diabetes. However, pediatric studies do not support a role of periodontal disease in the pathogenesis of T1D and its complications and exclude an effect on glycemic control.

The hypothalamic-pituitary-gonadal axis is discussed in two comprehensive reviews. Bizzarri and Cappa offer an interesting description of the transient sex-specific activation of hypothalamic-pituitary-gonadal axis during the first 6 months of life in boys, and the first 2 years in girls. This phenomenon, known as “mini-puberty,” is fascinating and provides insights into the understanding of the mechanisms of pubertal development later in life. Festa et al. review genetic causes of pubertal delay and propose a diagnostic algorithm to distinguish between self-limited delayed puberty and other forms of hypogonadotropic hypogonadism. Self-limited delayed puberty is a frequent condition in endocrine clinical practice, whereas congenital hypogonadotropic hypogonadism accounts for 4% and 5.7% cases of delayed puberty in boys and girls, respectively. The authors offer an in-depth discussion of the benefits and limitations of genetic testing in the clinical setting, underling its potential role for a better classification and management of delayed puberty.

Assessment of bone maturation (BM) in children and adolescents is relevant in medical and non-medical fields (5). Despite a few issues related to the use of different methods, bone age is a unique tool to assess the maturational changes during childhood and adolescence, and to aid the diagnostic workout of growth disorders. In this Research Topic, Cavallo et al. provide an overview of the main methods to assess bone age and discuss their advantages and disadvantages. In a second paper, Pepe et al. report the results of a prospective study assessing the link between BM and postnatal growth during the first year of life in a population of children born small for gestational age (SGA). Although most SGA children show spontaneous catch-up growth during the first two years of life, a percentage of them remains short during childhood (6). In the present study, SGA newborns with delayed BM showed greater growth velocities and height gains at 12 months of age compared to those with adequate BM at birth. Besides, the study shows that ultrasonographic evaluation of the Béclard’s nucleus could be a useful noninvasive technique to identify intrauterine BM delay.

Another key topic discussed in this issue is the reason for and appropriateness of pediatric endocrinology referrals. A clear understanding of referral pathways is essential to optimize resource use and plan continuous medical education and public health interventions. Bellotto et al. report data on endocrine referrals to a single Italian tertiary center for pediatric endocrinology. The study highlights that growth and puberty represent nearly half of all reasons for endocrine referrals, and that timing for referral (i.e. urgent vs deferrable priority) is often inappropriate. The authors suggest that medical education of primary care can be better tailored to improve the timing of referral and the ability to recognize subclinical and para-physiological conditions. These steps are paramount to better define windows of intervention and optimize clinical outcomes.

We hope that this Research Topic will provide a valuable resource for the management of clinical endocrine issues in daily practice and we encourage the readers to submit their General Commentary articles on these articles.

## Author Contributions

All authors listed, have made substantial, direct, and intellectual contribution to the work, and approved it for publication.

## Conflict of Interest

The authors declare that the research was conducted in the absence of any commercial or financial relationships that could be construed as a potential conflict of interest.
